# Bridging Continuous and Discrete Models of the Anterior Temporal Lobe via Cortical Gradients

**DOI:** 10.21203/rs.3.rs-9023856/v1

**Published:** 2026-03-24

**Authors:** Tirso Gonzalez Alam, Michel Thiebaut de Schotten, Richard Binney, Xiuyi Wang, Daniel Margulies, Beth Jefferies

**Affiliations:** Bangor University; Institut des Maladies Neurodégénératives-UMR 5293; Bangor University; Institute of Psychology, Chinese Academy of Sciences; University of York; University of York

**Keywords:** Anterior temporal lobe, DWI, tractography, cortical organisation, large-scale networks, semantic cognition, semantic memory, semantic control

## Abstract

The anterior temporal lobe (ATL) is critical for semantic cognition, yet its organisation remains debated. Some evidence supports a patchwork of discrete, domain-specific parcels, while the graded hub hypothesis proposes smooth, ordered transitions. We examined regions along ATL’s curvature, testing whether connectivity profiles varied systematically or discontinuously along whole-brain dimensions of functional connectivity. The structural and functional connectivity of ATL varied largely systematically on the heteromodal-unimodal dimension, with intermediate ventral parcels showing stronger heteromodal connectivity; however, medial parcels deviated from this pattern. On the auditory-motor to visual dimension, there were partial dorsal-to-medial shifts in connectivity, but parcels at both ends did not follow this trend. There were also systematic dorsal-to-medial connectivity shifts between default and control networks. Individual differences in structural connectivity predicted activation in auditory tasks. These results largely support a graded hub model of ATL, while also revealing non-systematic functional variation that may enable flexible semantic processing.

## 1. Introduction

Semantic cognition – the ability to extract and manipulate the meanings of words, objects and events – is fundamental for everyday behaviour. This capacity relies on two highly interactive components: the storage of rich, transmodal representations in semantic memory, and the controlled retrieval of goal- and context-relevant features from these representations ^1–3^. Converging evidence from lesion, neuroimaging, and neurostimulation studies implicates the bilateral anterior temporal lobe (ATL) as a central hub for semantic representations. The ATL integrates inputs from distributed sensorimotor “spokes” and from affective and language networks to form coherent, generalisable concepts ^4–13^.

Recent methodological advances – particularly optimised functional MRI (fMRI) sequences mitigating signal dropout and distortion in ventral and medial temporal ATL ^14–17^ – have enabled finer-grained investigation of this region’s organisation. These studies support the graded hub hypothesis, in which heteromodal integration emerges gradually along multiple input pathways: visual information enters via ventral and medial temporal regions, auditory information via superior temporal sulcus and gyrus, and convergence across these modalities peaks in anterior lateral and ventral temporal cortex ^13,18,19^. Under this view, semantic responses in ATL range from relatively modality-specific to fully heteromodal, with maximal integration occurring at the midpoint between the primary input streams ^1,14,20–22^.

Structural and functional connectivity as well as meta-analytic studies reveal systematic transitions in ATL function, supporting the view that there is graded organisation within this area. Studies show greater modality convergence in anterior versus posterior portions of ATL, while lateral and ventral regions, further from visual and auditory inputs streams, show stronger connectivity to default mode and limbic systems respectively^18,21,23,24^. For example, Binney et al. (2012) used tractography to demonstrate both anteroposterior and dorsoventral variation in structural connectivity, consistent with modality convergence along multiple axes (see also ^19,25^). Meta-analytic findings also show dorsolateral–ventromedial differences in function – in modality, linguistic abstraction, and socioemotional features^21,26^. The principle of graded organisation is also evident in patterns of intrinsic functional connectivity and in multimodal cortical parcellations, with parcels showing systematic shifts in function along the cortical surface ^23,27,28^. Toward the most anterior portion of the temporal lobes, auditory and visual streams merge into a less distinct but still highly organised representational space that retains echoes of dorsoventral specialisation ^27,29,30^.

In contrast, other lines of evidence support a patchwork view, in which the ATL is composed of discrete functionally specialised regions instead of gradually-varying responses. Portions of the ATL have been implicated in representing specific semantic domains, including living things and artifacts ^31–33^, unique entities such as proper names^34–36^, and abstract properties of objects ^36,37^. Other studies implicate ATL subregions in social and emotional processes ^38–42^ or in face processing ^43^. From this perspective, the ATL is not a unified hub, but a collection of domain-specific parcels embedded in distinct functional hierarchies, with sharp boundaries rather than smooth transitions ^44^.

This contrast raises a key question: does ATL organisation reflect smooth, graded and systematic transitions from unimodal to heteromodal processing^1,13,21,45,46^, or discrete functional parcels with sharp boundaries? Recent evidence hints that the answer may lie between these two extremes^47^. Krieger-Redwood et al. (2024), using optimised fMRI methods to maximise ATL signal, reported both gradual and sharp transitions depending on task and modality, suggesting that functional boundaries in the ATL are not fixed but context-dependent. This raises the possibility that structural organisation in the ATL might likewise reflect a mixture of continuous and discrete principles, providing the substrate for flexible functional recruitment. Specifically, while the underlying white matter tracts could provide the fixed scaffolding necessary for the implementation of relatively invariant transmodal representations in the ATL hub, the functional connectivity arising from this scaffolding would need to remain dynamic to meet varying task demands. In transmodal cortex, this could manifest as a divergence, or loose coupling of structural and functional principles of organisation, allowing a single structural architecture to support multiple, reconfigurable functional states.

To test this possibility, we examined the macroscale organisation of ATL by projecting its parcels’ patterns of structural connectivity into a whole-brain functional state space defined by connectivity gradients derived from resting-state fMRI. This allowed us to assess the connectivity of each parcel on dimensions capturing key functional distinctions across the cortex over time – and to ask whether neighbouring parcels show similar or different patterns of connectivity for each dimension. Connectivity gradients are defined using diffusion embedding (a nonlinear dimensionality reduction technique related to principal components analysis), which decomposes whole-brain functional connectivity into smooth axes which capture patterns of variation in connectivity that occur over time. The first gradient, explaining the most variance in resting-state fMRI, spans unimodal to heteromodal cortex, the second separates visual from auditory–motor systems, and the third distinguishes default mode from control networks ^48^. The principal gradient also correlates with cortical distance from primary sensory and motor cortex, reflecting a continuous processing hierarchy from perception to high-order integration. These gradients reveal large-scale organisational principles that dominate functional coupling at different times. Anchoring ATL connectivity within this framework provides a principled means of testing whether its organisation varies across parcels smoothly along each axis, as predicted by graded hub theories, or instead shows discontinuities, as predicted by patchwork accounts. Accordingly, we traced a spline along the curvature of the ATL in the Glasser et al. (2016) parcellation—spanning dorsolateral, ventral, and medial surfaces—and examined whether connectivity profiles changed in a systematic, graded manner or in a discontinuous, patchwork fashion.

**Figure 1.**
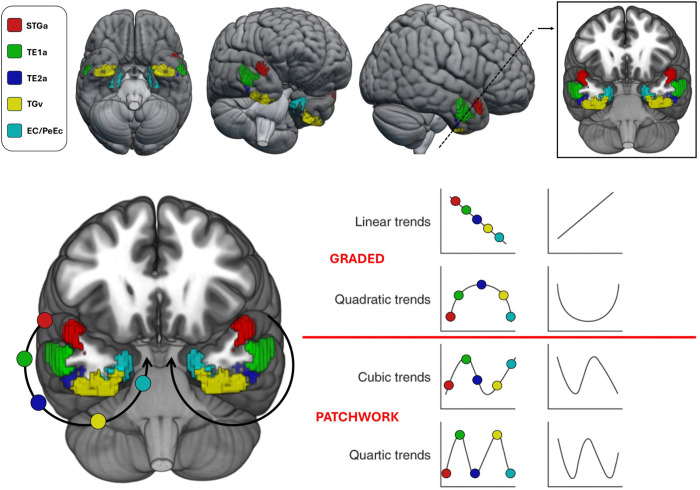
Regions of interest and illustration of hypothesis. Top row. Illustration of the Regions of Interest and their Glasser atlas labels. Bottom row. Illustration of the graded hub and patchwork framework predicted connectivity trends.

The graded hub hypothesis makes distinct predictions for each macroscale gradient (Fig. 1). Along the principal gradient, it predicts orderly quadratic transitions (“Quadratic trends”; Fig. 1): moving towards the middle of the spline from dorsolateral and medial extremes (red and cyan circles in Fig. 1), connectivity should shift from unimodal systems towards the transmodal “hub” (blue/yellow circles in Fig. 1) at the midpoint, producing a smooth inverted U-shaped pattern. Along the second gradient, the hypothesis predicts a linear transition from auditory to visual connectivity (“Linear trends”; Fig. 1) as we move from dorsolateral (red circle in Fig. 1) to ventral to medial regions (cyan circle in Fig. 1). Finding both quadratic (principal gradient) and linear (second gradient) trends would constitute strong evidence for graded organisation. In contrast, the patchwork hypothesis predicts higher-order (cubic, quartic) profiles, with connectivity shifting up and down along gradients in a discontinuous fashion, reflecting parcel-like mosaics rather than smooth transitions (see “Cubic trends” and “Quartic trends” illustration in Fig. 1). At the same time, analysis of ATL tract terminations on the third gradient addresses the question of whether specific parcels have stronger connectivity with executive control or default mode networks. This approach allows us to situate parcels of ATL within low-dimensional axes of whole-brain organisation, providing a scaffold for understanding how its functional responses might flexibly align with different large-scale systems over time, even though the underlying anatomical tracts are fixed. Finally, if structural connectivity patterns reflect functionally meaningful organisation, individual differences in their strength should covary with BOLD responses during linguistic tasks. We therefore examined whether connectivity-to-gradient mappings predicted task activations (maths and story comprehension) in the HCP dataset. To our knowledge, this is the first study to embed ATL structural connectivity in multidimensional cortical gradients, and to relate these patterns to functional activation in a large sample.

The results show that the structural connectivity of ATL parcels largely follows systematic, graded transitions when projected into macroscale gradient space. Specifically, connectivity along the principal gradient exhibited a U-shaped profile, with intermediate parcels showing the strongest heteromodal integration, consistent with the graded hub hypothesis. However, transitions along the second (visual-to-auditory) and third (control-to-default) gradients revealed specific regional deviations from this graded pattern. Furthermore, we demonstrate that individual differences in this structural scaffolding predict functional activation during linguistic tasks. We conclude that the ATL architecture combines a stable, graded axis of heteromodal integration with local, discrete variations that may support flexible, context-dependent semantic processing.

## 2. Results

### 2.1. Characterising the tracts

**Figure 2.**
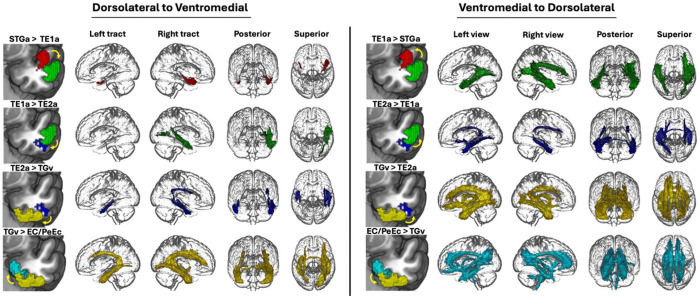
Results of sequential dorsolateral-to-medial contrasts between white matter tracts derived from seeding ATL ROIs. The tracts are colour-coded following the ROIs described in Figure 1. Note. STGa: Anterior division of the superior temporal gyrus, TE1a: Area TE1 anterior of Von Economo, TE2a: Area TE2 anterior of Von Economo, TGv: Area TG Ventral of Von Economo, EC / PeEc: Entorhinal and Perirhinal Ectorhinal Cortex.

To document the graded differences in the connectivity and function of the ATL from dorsolateral to ventromedial regions, we arranged our ROIs in a spline-like fashion, forming a semicircle from lateral dorsal ATL progressing ventrally to fusiform gyrus and terminating medially in the medial temporal lobe (See section 4.1.2 and Fig. 1). We treated those ROIs as seeds and extracted tracts for each of them. We then contrasted the white matter tract connectivity of parcels (i.e., seeds) that were adjacent to each other along this spline. The results can be seen in Fig. 2: multiple tracts are highlighted in these comparisons of adjacent parcels, including cingulum, inferior longitudinal fasciculus, fornix, uncinate and arcuate fasciculus. More medial parcels also show broader structural connectivity than dorsal parcels along the spline. Since displaying left and right hemisphere tracts in the same brain makes it difficult to appreciate fibres that cross over to the contralateral hemispheres, we repeated these plots displaying only one tract at a time to emphasize these crossing fibres in Supplementary Figures S2 – S4. The tracts derived from each parcel (i.e., not contrasting across parcels) can be consulted in Supplementary Figure S5.

### 2.2. Tract to gradient analysis

**Figure 3.**
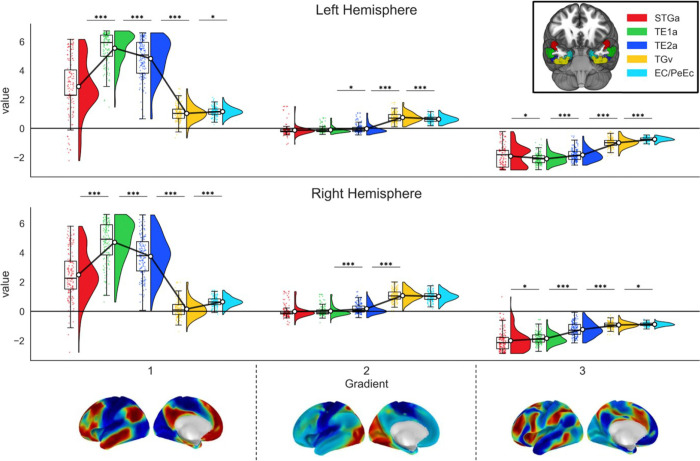
Results of the structural connectivity to cortical gradients analysis. The plot depicts the means and distributions of gradient values for tracts derived from each seed in the structural connectivity analysis. Data are visualized using raincloud plots: the half-violin plots illustrate the probability density distribution of the data, their accompanying box plots depict the median and interquartile range, and the dots represent individual data points. White circles with black outlines indicate the mean value for each seed, which are connected by solid black lines to illustrate the trend across regions. Note: STGa: Anterior division of the superior temporal gyrus, TE1a: Area TE1 anterior of Von Economo, TE2a: Area TE2 anterior of Von Economo, TGv: Area TG Ventral of Von Economo, EC / PeEc: Entorhinal and Perirhinal Ectorhinal Cortex. * p<.05, ** p<.01, *** p<.001. All p-values have been Bonferroni-corrected for multiple comparisons.

Next, we asked whether tracts for these seeds, derived from whole-brain tractography, showed systematic patterns in their terminations when projected into the whole-brain connectivity gradients described by Margulies et al^48^. These analyses examined the mean gradient value for all cortical hits for each seed for each participant (Fig. 3), which reflects the tract position in the space of that gradient. A tract positioned near the top of Gradient 1 space would exhibit stronger connectivity to heteromodal cortex, whilst unimodal cortex would be positioned near the bottom. The top of Gradient 2 represents visual cortex connectivity, with auditory/motor near the bottom. The top of gradient 3 represents cognitive control regions, with the bottom allied to the default-mode network. An omnibus analysis examined differences in these tract positions between parcels across hemispheres separately for each gradient. These analyses revealed two-way interactions of seed by hemisphere, as well as main effects of seed and hemisphere, although the left and right hemispheres showed broadly similar patterns (Table 1). We therefore performed our main analysis for each gradient separately for the left and right hemispheres.

Our key question was whether connectivity varies systematically within the ATL when comparing parcels along its curvature. To do this, we repeated the ANOVAs for each gradient and hemisphere separately, using Seed as a factor, and compared Estimated Marginal Means (EMM) between adjacent seeds. Since gradient 1 separates unimodal (low values) from heteromodal cortex (high values), and the graded hub hypothesis anticipates a heteromodal ventral hub sandwiched between the unimodal ends of the curvature of the ATL, we expected the seeds at the extremes of our spline to be lower in gradient 1 than those near the middle. For gradient 2, which separates auditory (lower values) from visual cortex (higher values), we expected that each parcel would show progressively higher values as we moved from STGa towards ventromedial ATL. For gradient 3, which distinguishes control from DMN regions, we did not make directional predictions but examined whether different ATL parcels preferentially align with one or the other system. The results can be seen in Table 2.

For gradient 1, we found evidence of the U shape as predicted, since the parcels towards the middle of our spline had the highest values (i.e. the most heteromodal connectivity) in both hemispheres. However, medial ATL parcels also showed a substantial difference from more lateral parcels – the transitions were not smooth. In addition, the most medial parcel was slightly but significantly more medial on gradient 1, which is not the pattern predicted by the graded hub model.

For gradient 2, there were some systematic shifts in connectivity from auditory-motor to visual between the three most lateral parcels in the middle of the spline, consistent with the predictions of the graded hub model. However, the most medial parcel was not more visual than the adjacent parcel (consistent with our observation that this parcel was also more heteromodal on gradient 1). There was also no difference between aSTG and the adjacent more lateral ATL parcel in either hemisphere. Therefore, only the middle portion of the spline meets the predictions of the graded hub model.

Gradient 3 showed fully systematic shifts in connectivity in the right hemisphere, with each successive pair of parcels showing a significant difference in the same direction: the most dorsal parcel in aSTG showed the strongest connectivity to DMN, while the most medial parcels showed more balanced connectivity to control and DMN ends of gradient 3. However, in the left hemisphere, aSTG was slightly more connected to control regions than the adjacent more lateral parcel.

Since our examination of each gradient revealed significant seed by hemisphere interactions for every seed, we also examined the differences between adjacent pairs of seeds across hemispheres for each gradient through a series of repeated measures ANOVAs. The results are provided in Supplementary Table S5. For gradient 1, one seed by hemisphere interaction was found, for TGv versus Ec/PeEc. For gradient 2, one seed by hemisphere interaction was found for TE2a versus TGv. For gradient 3, there were significant seed by hemisphere interactions for all comparisons. Left hemisphere tracts were consistently more heteromodal in Gradient 1, and right hemisphere tracts were more visual in gradient 2, in line with previous research ^49^. Gradient 3 exhibited the only cross-over interaction out of all comparisons examined for this study for the most dorsal pair of seeds (STGa vs TE1a).

### 2.3. Functional consequences of tracts relation to cortical hierarchy

Having established systematic structural differences in how our ATL seeds connect to the first three cortical gradients, we next asked whether these differences have functional consequences – specifically, whether structural variation between individuals predicts functional responses. To test this, we examined whether activation during two linguistic tasks in the HCP dataset – auditory semantic (story comprehension) and non-semantic (maths) – could be predicted from the connectivity strength of our seed regions. We used the seeds in two complementary ways: (1) as ROIs from which to extract task-related activation, and (2) as origins to quantify tract strength to gradient space. We then conducted two repeated-measures ANCOVAs, with task activation (Maths vs. Story) as factors and tract strength from each seed to each gradient as covariates (see section 4.1.3 for details).

**Figure 4.**
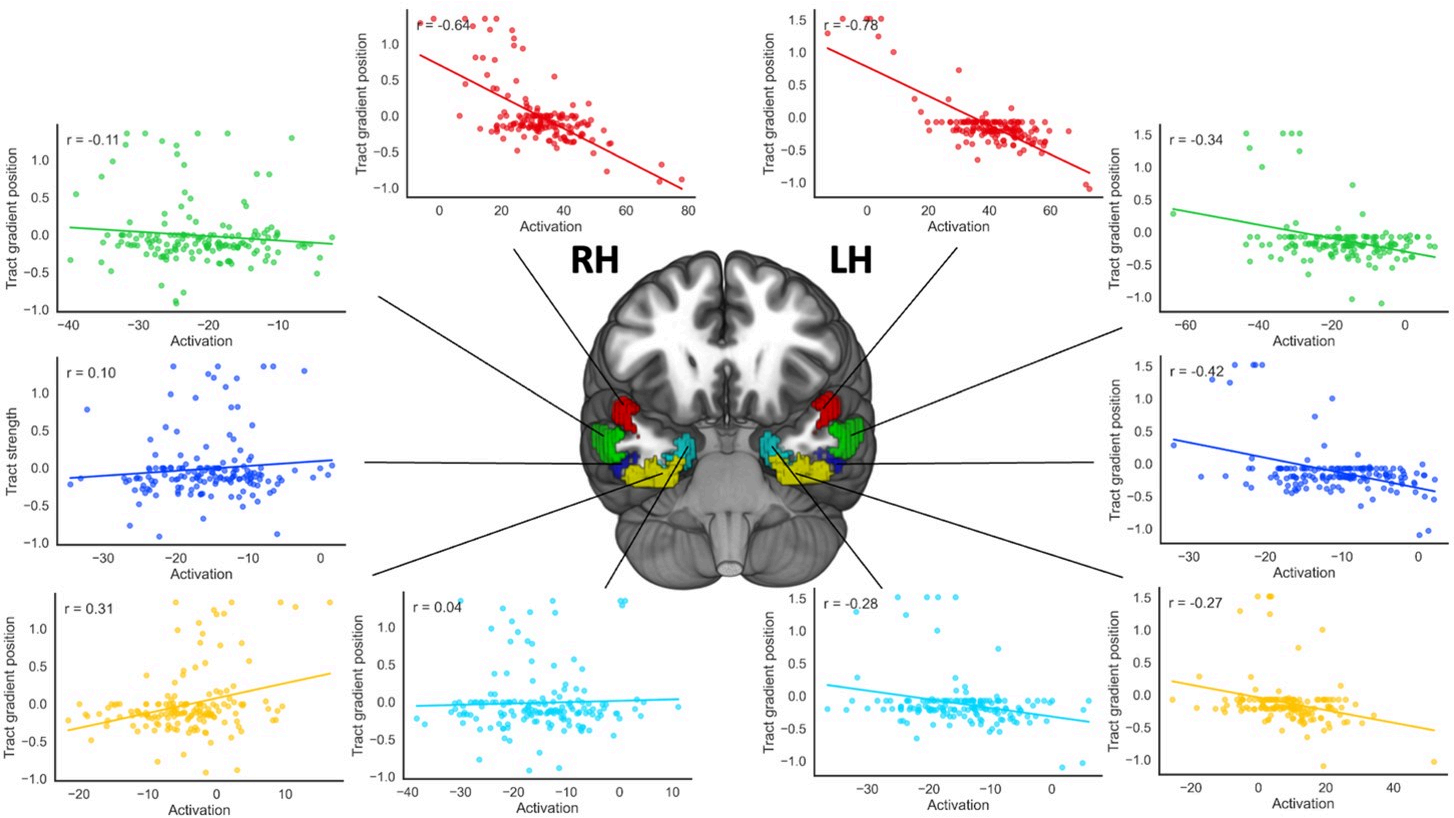
Results of the structural-functional association analysis for gradient 2. Scatterplots depicting the correlations of the beta values predicted by the ANCOVA model averaged across tasks within each ROI (x axis) with tract strength from the STGa ROI to gradient 2 (y axis). The plots corresponding to left and right hemisphere beta activations are connected through solid lines to their corresponding ROIs, and are colour-coded to match their corresponding ROI. Note: LH = Left Hemisphere, RH = Right Hemisphere.

The results revealed a bilateral interaction between the strength of connectivity of tract STGa to Gradient 2 with activation along the spline (left hemisphere: F(3.57,509.86) = 2.66, p=.038, η_p_^2^ = .018; right hemisphere: F(3.09,441.35) = 2.85, p=.036, η_p_^2^ = .02): activation across language tasks (story and maths) was strongly associated with connectivity from STGa to the auditory end of Gradient 2 in the most dorsal part of the spline, with the strength of this relationship decreasing in a linear fashion moving ventrally along the spline. This effect showed a linear trend in left hemisphere (F(1,143) = 4.55, p=.035, η_p_^2^ = .031) and a linear (F(1,143) = 4.25, p=.041, η_p_^2^ = .029) and quadratic (F(1,143) = 5.71, p=.018, η_p_^2^ = .038) pattern in right hemisphere, with the latter showing a larger effect size. Scatterplots visualising this interaction, which depict correlations of predicted beta values within each ROI plotted against mean Gradient 2 values for the tracts from the STGa ROI, are in Fig. 4. These results indicate that stronger connectivity to auditory–motor regions relative to visual regions is associated with greater activation to auditory inputs, particularly in aSTG. In right ATL, this relationship gradually decreases along the curvature and then reverses in medial regions, in line with the expectations of the graded hub account, whereas in left ATL the association is less systematic.

**Figure 5.**
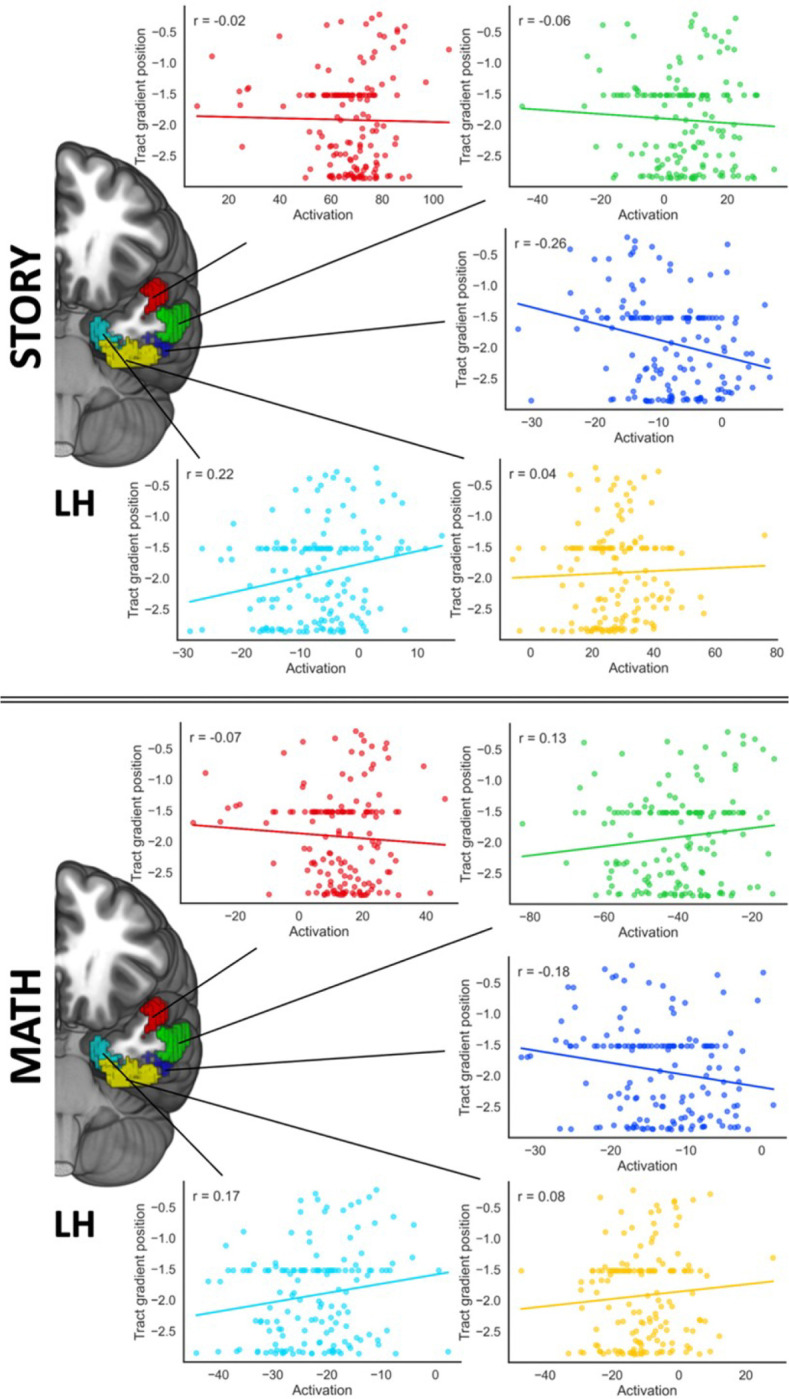
Results of the structural-functional association analysis for gradient 3. Scatterplots depicting the correlations of the beta values predicted by the ANCOVA model for the story (top panel) and math (bottom panel) tasks separately within each ROI (x axis) with tract strength from the STGa ROI to gradient 3 (y axis). The plots corresponding to each task beta activations for the left hemisphere are connected through solid lines to their corresponding ROIs, and are colour-coded to match their corresponding ROI. Note: LH = Left Hemisphere

We also observed a significant Task x ROI interaction with the strength of connectivity of tract STGa to Gradient 3 in the left hemisphere (F(3.51,501.16) = 2.94, p=.026, η_p_^2^ = .02). To interpret this interaction, we performed two follow-up ANCOVAs (one for each task) using ROI activation for the task in the left hemisphere as a factor, entering the strength of the connectivity of STGa to gradient 3 as a covariate. There was a significant quadratic association between the activation of the ROIs during the story task and the tract position of STGa in gradient 3 (F(1,143) = 5.1, p=.025, η_p_^2^ = .034). This quadratic trend was not replicated in a supplementary analysis excluding participants without cortical hits, and therefore must be interpreted with caution. No linear or quadratic relationships were detected for the activation of this region during the Maths task. The scatterplots depicting correlations between the predicted beta values for each ROI and mean gradient values for tracts from STGa to gradient 3 for each task can be consulted in Fig. 5. When STGa is more connected to the DMN end of Gradient 3, area TE2a shows more activity during the Story task, while area EC/PeEc shows less activity.

### 2.4. Resting-state replication

**Figure 6.**
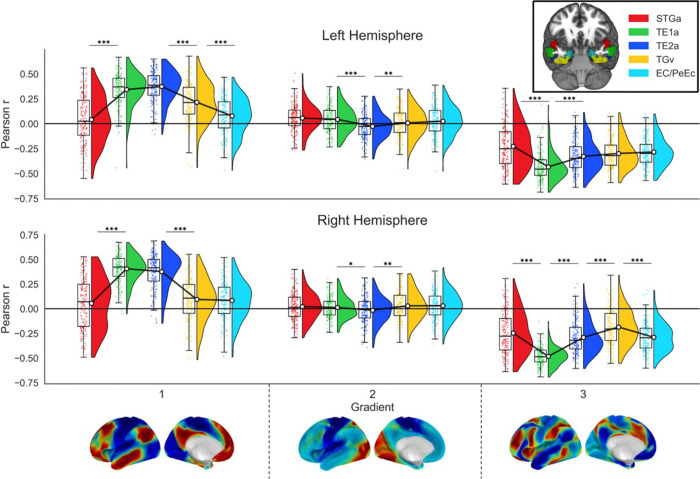
Results of the functional connectivity to cortical gradients replication analysis. The plot depicts the means and distributions of Pearson correlation values for seed-based correlation maps derived from each of our seeds with cortical gradient maps. Data are visualized using raincloud plots: the half-violin plots illustrate the probability density distribution of the data, their accompanying box plots depict the median and interquartile range, and the dots represent individual data points. White circles with black outlines indicate the mean value for each seed, which are connected by solid black lines to illustrate the trend across regions. Note: STGa: Anterior division of the superior temporal gyrus, TE1a: Area TE1 anterior of Von Economo, TE2a: Area TE2 anterior of Von Economo, TGv: Area TG Ventral of Von Economo, EC / PeEc: Entorhinal and Perirhinal Ectorhinal Cortex. * p<.05, ** p<.01, *** p<.001. All p-values have been Bonferroni-corrected for multiple comparisons.

To assess the robustness of our results, our final analysis investigated whether these patterns of connectivity were replicated using a different imaging approach in a separate sample. We conducted functional connectivity analysis in a separate 191-subject sample using resting-state data. We calculated the Pearson correlation (r) between each ROI seed-based correlation map per participant and the three gradient maps from Margulies et al. (2016), indicating the strength of connectivity of each ROI to each whole-brain gradient, and performed analyses like those used for the structural connectivity data. The results, which can be seen in Tables 3–4 and Fig. 6 largely replicated the pattern for gradient 1 observed above. The patterns of connectivity followed the predictions of the graded hub account, since the most heteromodal regions were in the middle of the spline, with the more dorsal and medial regions showing more unimodal connectivity. The TE1a and TE2a ROIs were the most heteromodal in both hemispheres, with TE1a being significantly more heteromodal than STGa and TE2a significantly more than TGv. In the left hemisphere TGv was significantly more heteromodal than EC/PeEC, but not in the right hemisphere. The results for gradients 2 and 3, on the other hand, did not show a clear replication of the patterns seen in structural connectivity. For gradient 2, there was some evidence of increasing connectivity to the visual end of the gradient between ventral and medial seeds, but the lateral/dorsal seeds did not exhibit a similar pattern for auditory-motor connectivity. In line with the structural connectivity results, gradient 3 showed a strong alliance of TE1a with the DMN, and some evidence of a systematic shift away from DMN across ROIs located in dorsal (STGa) to medial (TE2a) regions along the spline, but this was only significant for two of the four contrasts in both hemispheres.

## 3. Discussion

Using a spline along the dorsal-to-medial curve of the anterior temporal lobe (ATL), we examined whether structural and functional connectivity varies systematically across adjacent parcels (consistent with a graded hub) or discontinuously (consistent with a patchwork organisation). Connectivity profiles were projected into macroscale cortical gradients derived from diffusion embedding, capturing low-dimensional axes of whole-brain organisation. Gradient 1 spans unimodal to heteromodal cortex, gradient 2 separates auditory–motor from visual systems, and gradient 3 distinguishes default mode from control networks. Structural connectivity largely varied systematically along the spline on these axes: middle portions of the ATL exhibited the strongest heteromodal connectivity on gradient 1, while dorsal-to-medial regions on gradient 3 showed transitions from default mode to control network connectivity. Functional connectivity showed similar patterns for the unimodal-to-transmodal axis captured by gradient 1, and individual differences in structural connectivity predicted task-related activation during auditory semantic and non-semantic tasks. Together, these results support a partially graded hub view of the ATL, in which structural architecture constrains functional responses, while deviations from smooth transitions allow for flexible, context-dependent recruitment of specific subregions.

In our tractography analysis, Gradient 1 showed a partial U-shaped pattern: dorsal and ventral extremes were less heteromodal than middle parcels, consistent with a graded hub in which multimodal integration can occur in cortical regions that are juxtaposed between auditory and visual processing streams ^1,18,21^. However, the most medial parcel encompassing entorhinal and perirhinal cortex was somewhat more heteromodal than predicted. This finding is consistent with the view that medial temporal cortex adjacent to the hippocampus supports high-level conceptual knowledge of specific entities, rather than only visual aspects of knowledge ^50,51^, especially the anterior portions of this structure, consistent with our seed. Gradient 2 provided weaker evidence for systematic connectivity shifts: some lateral and ventral parcels followed the predicted auditory–motor to visual progression, but medial parcels and aSTG did not. Gradient 3 revealed largely systematic shifts from dorsal to medial ATL: connectivity was biased towards DMN in dorsal parcels, and shifted towards the middle of this control-DMN axis in more medial parcels. However, left aSTG showed less DMN connectivity than expected from this pattern, consistent with its involvement in language processing ^52,53^, which requires access to both memory representations in DMN and control processes that can allow task- and context-relevant information to be prioritised^54^. A parallel analysis of intrinsic connectivity from resting-state fMRI data for each parcel from an independent sample largely confirmed these effects for gradient 1, but gradients 2 and 3 showed less consistency between structural and functional connectivity. These deviations may reflect local functional specialisation or context-dependent recruitment, in line with recent work reporting both smooth and sharp functional transitions in the ATL ^47^.

Individual differences in structural connectivity also predicted functional recruitment. In aSTG, stronger tracts to auditory–motor regions along gradient 2 were associated with increased activation to auditory inputs, particularly in the left hemisphere, with this effect gradually declining and even reversing toward medial ATL. Medial parcels responded more strongly when aSTG was more connected to the visual end of gradient 2, possibly reflecting enhanced input via the ventral visual stream. Connectivity of aSTG with the DMN end of gradient 3 predicted differential activation across other ATL regions during story comprehension: when aSTG was more connected to DMN regions, lateral ATL parcels showed increased activation to the spoken story, while entorhinal cortex showed reduced activation. This pattern suggests that the influence of aSTG connectivity on functional responses is region-specific, potentially reflecting the ATL subregions that receive the most relevant input under specific task conditions.

A key finding was the distinction between the principal gradient, which was replicated across structural and functional connectivity, and the secondary gradients, which showed divergence between structure and function. This pattern of results is compatible with the proposal that gradient 1 acts as a stable axis of organisation – spanning unimodal to heteromodal cortex – implemented in architectural constraints, whilst simultaneously allowing alignment with specific sensorimotor or control systems on secondary gradients in a more flexible fashion. According to this view, macroscale gradients act as a coordinate system where the principal axis anchors stable structural connections needed for invariant representations, while the secondary axes capture the dynamic functional shifts required for specific tasks. Thus, ATL is structurally wired as a graded hub (gradient 1), but functionally retains the capacity to shift between modalities (gradient 2) and control networks (gradient 3). Structure-functional decoupling is a hallmark of transmodal cortex ^55^, reflecting the need to generate diverse functional states to support flexible cognition ^56,57^. Our results also show that structural connectivity is predictive of brain activation during a task, suggesting that the underlying anatomical scaffolding, while flexible, directly constrains and enables context-specific functional responses.

Several limitations of this study should be considered. Our spline-based approach focused on the anterior-most aspects of the temporal lobe, anatomically defined by the Harvard-Oxford atlas, expected to be critical for auditory-visual integration, but parcels were in highly heteromodal cortex and showed low variability in connectivity along the second gradient. Stronger effects may emerge in slightly more posterior regions where sensory input streams are more apparent. In addition, comparing our parcels to studies observing semantic activation with task fMRI reveals that our parcels are anterior to some previously reported peaks ^22,46^. Thus, a single spline within ATL may not be sufficient to capture all the functional transitions occurring in this brain region. Second, our analysis relied on deterministic tractography, which can have difficulty resolving complex fibre crossings; future work could validate these findings with probabilistic algorithms. Finally, while the gradients provide a powerful, data-driven framework to summarise whole-brain organisation, they are themselves derived from functional connectivity, and the interpretation of structural projections onto this space is an emerging area of research.

Overall, the data largely support a graded hub framework in ATL: systematic yet not necessarily smooth transitions dominate, particularly in heteromodal middle regions of the spline reflecting a primary, graded axis of information convergence. At the same time, local deviations and region-specific structural-functional dissociations hint to a system sufficiently nuanced to support functionally specialised nodes that can drive different states over time, reconciling continuous and discrete models of ATL organisation. This architecture provides a foundation for an essential aspect of semantic cognition: the ability to integrate modality-specific inputs into a vast store of knowledge while retaining flexibility to adapt recruitment according to task demands, thus enabling their retrieval in a flexible, context-dependent manner.

## 4. Methods

### 4.1. DWI

The data used in this study were obtained from the Human Connectome Project (HCP). The original HCP data collection protocol was approved by the Washington University in St. Louis Institutional Review Board (IRB). Informed consent was obtained from all participants by the original Human Connectome Project investigators.

#### 4.1.1. DWI pre-processing

We used data from a subgroup of 164 HCP participants who underwent diffusion-weighted imaging at 3 Tesla^58^ (http://www.humanconnectome.org/study/hcp-young-adult/). The imaging parameters were previously described in Uǧurbil et al. 2013 and involved acquiring 111 near-axial slices with an acceleration factor of 32, an isotropic resolution of 1.25 mm^3^, and coverage of the entire head. The diffusion-weighted images were obtained using 90 uniformly distributed gradients in multiple Q-space shells ^59^, and this process was repeated three times with different b-values and phase-encoding directions. We used a pre-processed version of this dataset, previously described ^60–62^, that included steps to correct for susceptibility-induced off-resonance field, motion, and geometrical distortion.

We used StarTrack software (https://www.mr-startrack.com) to perform whole-brain deterministic tractography in the native DWI space. We applied an algorithm for spherical deconvolutions (damped Richardson-Lucy), with a fixed fibre response corresponding to a shape factor of α = 1.5 × 10–3 mm2.s – 1 and a geometric damping parameter of 8. We ran 200 algorithm iterations. The absolute threshold was set at three times the spherical fibre orientation distribution (FOD) of a grey matter isotropic voxel, and the relative threshold was set at 8% of the maximum amplitude of the FOD ^63^. To perform the whole-brain streamline tractography, we used a modified Euler algorithm ^64^ with an angle threshold of 45°, a step size of 0.625 mm, and a minimum streamline length of 15 mm.

To standardize the structural connectome data, we followed these steps: first, we converted the whole-brain streamline tractography into streamline density volumes, with the intensity corresponding to the number of streamlines crossing each voxel. Second, we generated a study-specific template of streamline density volumes using the Greedy symmetric diffeomorphic normalization pipeline provided by ANTs ^65^. This average template was created for all subjects. Third, we co-registered the template with a standard 1mm MNI152 template using the FLIRT tool in FSL to produce a streamline density template in the MNI152 space. Finally, we registered individual streamline density volumes to the template and applied the same transformation to the individual whole-brain streamline tractography using ANTs GreedySyn and the Trackmath tool in the Tract Querier software package ^66^. This produced whole-brain streamline tractography in the standard MNI152 space.

#### 4.1.2. Regions of interest

Since our main hypothesis concerns systematic variation as we traverse the ATL, we selected a series of parcels from the STG, then traversing the lateral face, passing through the ventral surface before terminating in the medial aspect (e.g., parahippocampal complex). Parcels were selected using the atlas of Glasser and colleagues^23^, which has two key advantages: (i) parcels are derived from multiple sources of information, including cytoarchitecture, functional connectivity and cortical folding patterns, helping to ensure the parcellation captures functionally-relevant regions despite signal drop out in MRI of the ATL; (ii) the parcellation identifies homotopes of parcels across both hemispheres, allowing their connectivity to be compared. We considered all parcels within the ATL in each hemisphere as defined by the Harvard-Oxford atlas, specifically Temporal Pole (TP, region 8), the anterior divisions of Superior, Middle and Inferior Temporal Gyri (STGa, MTGa, ITGa, regions 9, 11 and 14 respectively), the anterior division of Parahippocampal Gyrus (PhGa, region 34) and the anterior divisions of Temporal Fusiform Cortex (TFCa, region 37). Within this broad anatomical mask, we first identified all parcels that contained more than 1000 voxels within the ATL. We then identified sequential Glasser parcels along a spline following the dorsolateral to ventromedial curvature of ATL (Fig. 1).

To test our hypothesis of systematic variation along a cortical trajectory on the surface of the ATL, we required a continuous set of parcels. However, the combined parcel size and anatomical mask constraints produced a gap in ITG. We therefore included an additional ITG parcel by relaxing the 1000-voxel threshold (Supplementary Table S1). We validated this choice by showing that tracts derived from the additional parcel had tract volumes and gradient variance comparable to those of adjacent, larger parcels (see Supplementary Analysis 1 and Supplementary Figure S1). The final parcels, which can be seen in Fig. 1, were centred on STG (Glasser parcel STGa), MTG (Glasser parcel TE1a), ITG (Glasser parcel TE2a), inferior temporal polar cortex / anterior fusiform gyrus (Glasser parcel TGv), and medial structures (combining perirhinal ectorhinal and entorhinal cortex, Glasser parcels EC and PeEc).

Although the dorsal component of the temporal pole (Glasser parcel TGd) met the size criterion, it was excluded to maintain topological and functional consistency with the spline-based analysis. Topologically, the parallel gyral architecture (superior, middle, and inferior temporal gyri) used to define the spline converges at the temporal pole^29^, making it difficult to define a continuous trajectory that is comparable to neighbouring parcels along the spline. Functionally, prior work indicates that anterior aspects of ventral and lateral temporal lobe regions (captured here by parcels TGv, TE1a, and TE2a) form a clearer locus of heteromodal semantic convergence^14,22^, whereas the organisation of more dorsal temporal pole regions is less well characterised in terms of systematic gradients or trajectories^27,30^.

Since parcels can be different shapes in the left and right hemispheres, we projected the left hemisphere parcels into the right hemisphere and identified the overlap. These anatomically identical regions could then be compared between left and right ATL, allowing us to assess if there are systematic patterns of variation across hemispheres. Figure 1 and Supplementary Table S1 provide details for these ROIs. Supplementary Table S1 contains the Glasser atlas ID and label of each ROI, the anatomical region label offered in the atlas, its centroid in MNI space (reported in left hemisphere space), its total volume, and a segmentation of this volume in grey and white matter for each of our ROIs^1^. These ROIs can be consulted in Neurovault^67^ (collection link: https://neurovault.org/collections/HOEXBXOG).

#### 4.1.3. Tract extraction and analytical approach

Our starting point for the tract extraction was each participant’s whole-brain streamline tractography in MNI (1mm) space, using the parcels described in section 4.1.2. as ROIs. For each of our ROIs, we used trackvis^68^ to extract all streamlines emerging from these regions as a volume in each hemisphere separately, yielding one streamline group (tract) per seed per hemisphere per participant.

Firstly, in order to characterise the spatial topography of the tracts derived from each of our seeds at the group level, we submitted all participants’ tracts for each seed to a one-way t test permutation analysis using FSL randomise^69,70^ (5,000 permutations with Threshold-Free Cluster Enhancement), thresholding the results at p < .01. We also produced contrasts between each adjacent pair of seeds along the spline (i.e., STGa > TE1a and the reverse) as part of this group level analysis. The resulting tracts were used to produce Figs. 2 and S2-S5, and can be consulted in Neurovault^67^ (collection link: https://neurovault.org/collections/HOEXBXOG).

Our main analysis aimed to address the question of systematic vs patchwork transitions in connectivity profiles across the ATL. To do this, we focussed on characterising the position of each tract of adjacent seeds in the three dimensions of whole-brain intrinsic connectivity that explain the most variance in the human cortex^48^. These dimensions are often referred to as “gradients” and are obtained using the technique of diffusion embedding to decompose whole-brain connectivity into its key components. They afford the opportunity to analyse the connectivity of the ATL in an ROI-free (i.e., whole-brain), multidimensional information-rich space and are thus able to capture complex and subtle patterns that might escape other methods.

We related tract terminations to Gradient 1, which captures the separation of heteromodal and unimodal cortex, Gradient 2, which separates visual from auditory-motor cortex, and Gradient 3, which separates control networks from DMN. To this end, we computed the overlap of each tract with these three gradients at the participant level multiplying a binarised mask of each tract per participant with each of the three gradient maps for each hemisphere. For each of these multiplications, we then extracted the mean value of the cortical hits into the gradient map. Some individuals exhibited tracts that did not reach the cortex for parcels STGa (18% of tracts) and TE1a (6.6% of tracts). For these participants, it was not possible to compute the mean gradient value of these tract terminations, and we imputed these with the group mean gradient value for those tracts in each hemisphere. We performed a supplementary analysis to ensure that imputing the missing tracts in these parcels did not bias the results, by re-running the analysis steps below excluding cases with missing values altogether. The results were broadly replicated, as can be seen in Supplementary Analysis 2.

We entered these data into a series of repeated measures ANOVAs to explore whether there were ordered or haphazard transitions in connectivity as we traverse the curvature of the ATL, as assessed in terms of the average gradient values of the tract terminations at the cortex. First, we ran an omnibus repeated measures ANOVA using Seed, Gradient and Hemisphere as a factor. Where interactions were found, we performed follow-up ANOVAs for each level of the factor. Since gradient 1 separates unimodal (at the bottom) from heteromodal cortex (at the top), and the graded hub hypothesis proposes a heteromodal ventral hub sandwiched between the unimodal ends of the curvature of the ATL, we expected the seeds at the extremes of our spline to be lower in gradient 1 space than those near the middle, which should become gradually more heteromodal (i.e., closer to the top of the gradient). For gradient 2, which separates auditory (at the bottom) from visual cortex (at the top), we expected that each parcel along our spline would show higher values than the previous one moving from STGa towards ventral and medial ATL in gradient 2. If, on the other hand, the ATL exhibits a patchwork organisation we would expect transitions in connectivity to not follow graded patterns (i.e., linear or quadratic trends) but instead to exhibit cubic and higher-order trends.

To explore whether these patterns of variation in connectivity had functional consequences, we next investigated whether tract strength predicts activation in auditory semantic (stories) and non-semantic (maths) linguistic tasks. For this purpose, we ran two repeated measures ANCOVAs using the five ATL regions as ROIs and extracted activation in the Math and Story tasks within the HCP dataset^58^ (www.humanconnectome.org/study/hcp-young-adult/). We included Task and ROI as factors (2×5) examining BOLD responses in the left and right hemispheres separately. The covariates were the gradient values for tracts derived for each of our seeds in the same hemisphere (i.e. 15 covariates: 3 gradients by 5 seeds). We focussed on results showing significant interactions with covariates. If these results showed higher-order interactions with task (i.e., Task x ROI x Covariate), we interpreted them by performing two follow-up ANCOVAs in the relevant hemisphere (i.e., if the effect was observed in the left hemisphere, only that hemisphere was analysed). These ANCOVAs looked at each task separately and included ROI activation as a main effect and the same covariates as before. To clarify whether structural-functional associations exhibited ordered or haphazard transitions across the ATL, we also performed a planned contrast trend analysis in these follow-up ANCOVAs to determine the nature of the association between the connectivity covariate across the ROIs: this identified whether variation along the spline was linear, quadratic, cubic or quartic.

### 4.2. Resting-State Analysis

#### 4.2.1. Participants

One hundred and ninety-one student volunteers (mean age = 20.1 ± 2.25 years, range 18–31; 123 females) with normal or corrected-to-normal vision and no history of neurological disorders participated in this study. Written informed consent was obtained from all subjects prior to the resting-state scan. The study was approved by the ethics committees of the Department of Psychology and York Neuroimaging Centre, University of York. This data has been used in previous studies to examine the neural basis of memory and mind-wandering, including region-of-interest based connectivity analysis and cortical thickness investigations ^71–81^.

#### 4.2.2. Pre-processing

Pre-processing and statistical analyses of resting-state data were performed using the CONN functional connectivity toolbox V.20a (http://www.nitrc.org/projects/conn; ^82^ implemented through SPM (Version 12.0) and MATLAB (Version 19a). For pre-processing, functional volumes were slice-time (bottom-up, interleaved) and motion-corrected, skull-stripped and co-registered to the high-resolution structural image, spatially normalized to the Montreal Neurological Institute (MNI) space using the unified-segmentation algorithm, smoothed with a 6 mm FWHM Gaussian kernel, and band-passed filtered (.008 – .09 Hz) to reduce low-frequency drift and noise effects. A pre-processing pipeline of nuisance regression included motion (twelve parameters: the six translation and rotation parameters and their temporal derivatives), scrubbing (outlier volumes were identified through the composite artifact detection algorithm ART in CONN with conservative settings, including scan-by-scan change in global signal z-value threshold = 3; subject motion threshold = 5 mm; differential motion and composite motion exceeding 95% percentile in the normative sample) and CompCor components (the first five) attributable to the signal from white matter and CSF ^83^, as well as a linear detrending term, eliminating the need for global signal normalization ^84,85^.

#### 4.2.3. Seed Selection and Analysis

The seeds used to calculate intrinsic connectivity were binarised masks of the same five parcels described in section 4.1.2. (see also Supplementary Table S1) and depicted in Fig. 1. We excluded all non-grey matter voxels that fell within these masks before using them as seeds. We performed seed-to-voxel analyses convolved with a canonical haemodynamic response function (HRF) for each of these seeds.

To situate the position of our seeds in the whole-brain cortical hierarchy summarised by the three gradients, we calculated the whole-brain seed-based correlation map for each individual per ROI. Then, we correlated these spatial maps with the first three gradient maps from Margulies and colleagues^48^, yielding a Pearson r correlation coefficient per seed, per participant. These coefficients were entered into ANOVAs using seed, Hemisphere and Gradient as factors as described above. Lastly, to produce spatial maps at the group level, analyses were carried out using CONN with cluster correction at p < .05, and a threshold of p-FDR = .001 (two-tailed) to define contiguous clusters.

### 4.3. Statistics and Reproducibility

Statistical analyses of structural and functional connectivity data were performed using SPSS version 28 and MATLAB 2019a.

Sample Size and Data Exclusion: The structural connectivity analysis included a sample of 164 participants from the Human Connectome Project (HCP). The replication analysis using resting-state functional MRI included an independent sample of 191 participants. No statistical methods were used to pre-determine sample sizes, but our sample sizes are similar to or larger than those reported in previous publications ^49,86–88^. Five participants were excluded from the analysis reported in section 2.3 (*Functional consequences of tracts relation to cortical hierarchy)* due to missing task BOLD data. No other data were excluded from the analysis.

Statistical Tests and Corrections: Group-level tractography comparisons employed non-parametric permutation testing (5,000 permutations) using FSL’s *randomise* tool, with Threshold-Free Cluster Enhancement (TFCE) corrected at p < 0.01. Analysis of gradient values involved repeated measures ANOVAs and ANCOVAs. The assumption of sphericity was assessed, and Greenhouse-Geisser corrections were applied where violations occurred. Post-hoc pairwise comparisons were corrected for multiple comparisons using the Bonferroni method. For resting-state seed-based analysis, cluster-level correction was applied at p < 0.05 with a voxel-level threshold of p < 0.001 (FDR).

Reproducibility: To ensure robustness of the tract-to-gradient analysis, parallel analyses were conducted using structural connectivity and resting-state functional connectivity. Supplementary analysis excluded participants with no cortical hits in tract tracing (rather than imputing means), which replicated the main findings.

## Supplementary Material

Supplementary Files

This is a list of supplementary files associated with this preprint. Click to download.

• SUPPLEMENTARYMATERIALS.docx

## Figures and Tables

**Table 1:** Results of the repeated measures Seed x Hemisphere ANOVAs for the mean gradient values of the tracts projected to each of the three gradient maps.

Effect		df	F	p	η_p_^2^
Gradient 1	**Seed**	2.97, 468.79	1094.25	< .001 ^+^	.87
	**Hemisphere**	1, 158	156.5	< .001	.50
	**Seed x Hemisphere**	2.49, 394.09	4.33	.008^+^	.03
Gradient 2	**Seed**	3.75, 592.59	682.74	< .001 ^+^	.81
	**Hemisphere**	1, 158	207.6	< .001	.59
	**Seed x Hemisphere**	3.36, 530.27	11.36	< .001 ^+^	.07
Gradient 3	**Seed**	2.51,396.85	578.38	< .001 ^+^	.79
	**Hemisphere**	1, 158	37.01	< .001	.19
	**Seed x Hemisphere**	2.23, 351.56	38.98	< .001 ^+^	.20

Note: +- Greenhouse-Geisser corrected to address a violation of the assumption of sphericity.

**Table 2:** Planned contrasts of the estimated marginal means of adjacent seeds' gradient values for tracts projected to each of the three gradient maps.

Contrast	Left Hemisphere		Right Hemisphere	
	Mean Difference	p	Mean Difference	p
Gradient 1				
STGa - TE1a	−2.65	< .001	−2.22	< .001
TE1a - TE2a	.714	< .001	.964	< .001
TE2a - TGv	3.79	< .001	3.59	< .001
TGv - EC/PeEc	− .127	.026	− .5	< .001
Gradient 2				
STGa - TE1a	− .024	> .5	− .037	> .5
TE1a - TE2a	− .078	.01	− .157	< .001
TE2a - TGv	− .781	< .001	− .894	< .001
TGv - EC/PeEc	.122	< .001	.057	.39
Gradient 3				
STGa - TE1a	.176	.015	− .153	.034
TE1a - TE2a	− .264	< .001	− .613	< .001
TE2a - TGv	− .845	< .001	− .304	< .001
TGv - EC/PeEc	− .24	< .001	− .044	.043

All p-values have been Bonferroni-corrected for multiple comparisons. Note: STGa: Anterior division of the superior temporal gyrus, TE1a: Area TE1 anterior of Von Economo, TE2a: Area TE2 anterior of Von Economo, TGv: Area TG Ventral of Von Economo, EC / PeEc: Entorhinal and Perirhinal Ectorhinal Cortex.

**Table 3:** Results of the repeated measures Seed x Hemisphere ANOVAs for the Pearson r values of the seed-based correlation maps correlated with each of the three gradient maps.

Effect		df	F	p	η_p_^2^
Gradient 1	**Seed**	3.12, 593.43	236.427	< .001 ^+^	.55
	**Hemisphere**	1, 190	.782	.378	.00
	**Seed x Hemisphere**	3.31, 629.67	19.53	< .001 ^+^	.09
Gradient 2	**Seed**	3.45, 664,72	11.37	< .001 ^+^	.06
	**Hemisphere**	1, 190	1.09	.298	.01
	**Seed x Hemisphere**	3.79, 719.27	7.47	< .001 ^+^	.04
Gradient 3	**Seed**	3.18, 604.35	110.9	< .001 ^+^	.37
	**Hemisphere**	1, 190	6.35	.013	.03
	**Seed x Hemisphere**	3.17, 603.03	22.77	< .001 ^+^	.11

Note: +- Greenhouse-Geisser corrected to address a violation of the assumption of sphericity.

**Table 4:** Planned contrasts of the estimated marginal means of adjacent seeds' Pearson r correlation values for seed-based correlation maps with each of the three gradient maps.

Contrast	Left Hemisphere		Right Hemisphere	
	Mean Difference	p	Mean Difference	p
Gradient 1				
STGa - TE1a	− .3	< .001	− .35	< .001
TE1a - TE2a	− .03	.11	.029	.124
TE2a - TGv	.158	< .001	.282	< .001
TGv - EC/PeEc	.137	< .001	.01	> .5
Gradient 2				
STGa - TE1a	.013	> .5	.009	> .5
TE1a - TE2a	.07	< .001	.026	.016
TE2a - TGv	− .035	.005	− .044	.003
TGv - EC/PeEc	− .018	> .5	− .001	> .5
Gradient 3				
STGa - TE1a	.206	< .001	.237	< .001
TE1a - TE2a	− .105	< .001	− .192	< .001
TE2a - TGv	− .029	.097	− .105	< .001
TGv - EC/PeEc	− .014	> .5	.104	< .001

All p-values have been Bonferroni-corrected for multiple comparisons. Note: STGa: Anterior division of the superior temporal gyrus, TE1a: Area TE1 anterior of Von Economo, TE2a: Area TE2 anterior of Von Economo, TGv: Area TG Ventral of Von Economo, EC / PeEc: Entorhinal and Perirhinal Ectorhinal Cortex.

## Data Availability

The diffusion data used in this study are publicly available from the Human Connectome Project (HCP) database (https://db.humanconnectome.org). Users must agree to the HCP Open Access Data Use Terms to access the dataset. The functional connectivity data used in this study can be accessed via written request to the ethics committee at the York Neuroimaging Centre.

## Footnotes

1. We classified each voxel as grey or white matter using the Harvard-Oxford Cortical Atlas as a starting point, assuming that everything not contained in it is sub-cortical (white), and everything in it is cortical (grey). Since this atlas does not track the sulci and gyri accurately, and there is some overlap between grey and white matter voxels we dilated the atlas with 1 extra layer of voxels using fslmaths, and classified those voxels as Grey/White border, since their inclusion in either category could be equivocal.

## Code availability

Whole-brain deterministic tractography was performed using StarTrack software (https://www.mr-startrack.com). Diffusion geometry processing and template generation were conducted using ANTs (https://github.com/ANTsX/ANTs) and the Tract Querier package (https://github.com/demianw/tract_querier). Functional connectivity preprocessing and analysis used the CONN toolbox version 20a (http://www.nitrc.org/projects/conn) and SPM12 (https://www.fil.ion.ucl.ac.uk/spm/), running in MATLAB 2019a. Statistical analyses were performed using SPSS v28 (statistical analysis). The custom scripts used to project tract terminations into gradient space and perform the statistical comparisons reported in this study are available from the corresponding author upon request.
